# Spatial accessibility of substance use disorder treatment programs, compared with other health care facilities, in New York State, 2024

**DOI:** 10.1186/s13722-025-00592-9

**Published:** 2025-07-31

**Authors:** Marcus A. Bachhuber, Chinazo O. Cunningham, Pat Lincourt, Ashly E. Jordan

**Affiliations:** 1 New York State Office of Addiction Services and Supports, 501 7Th Avenue, New York, NY 10018 USA; 2 Department of Medicine, Oregon Health & Science University, Portland, OR USA

**Keywords:** Spatial accessibility, Substance use disorder, Outpatient treatment, Residential treatment, Opioid treatment programs

## Abstract

**Background:**

Spatial accessibility of substance use disorder (SUD) treatment is a crucial component of access and a comprehensive analysis can help to identify if and where a lack of spatial accessibility is a barrier to treatment.

**Methods:**

We conducted a cross-sectional analysis of spatial accessibility of SUD treatment (outpatient, opioid treatment program, and residential) in New York State (NYS). We estimated two measures of spatial accessibility: one-way travel time (i.e., drive time for NYS outside of New York City [NYC] and public transit time for NYC) and robustness (i.e., the difference in travel time between the closest and fifth closest facility). Comparison facilities included Federally Qualified Health Centers, dialysis facilities, and hospitals. We compared travel time and robustness by urbanicity (NYC, urban non-NYC, rural) and NYS economic development region using population-weighted paired t-tests.

**Results:**

The percentage of NYS residents within 30 min travel time was 97.2% for outpatient SUD treatment programs and 82.3% for opioid treatment programs. Mean statewide travel time to outpatient SUD treatment programs was comparable to travel time to Federally Qualified Health Centers (difference: 1.0 min [95%CI 0.9 to 1.1; *P* < 0.001]) and dialysis facilities (difference: 0.1 min [95%CI 0.03 to 0.2; *P* = 0.01]), and significantly shorter than to hospitals (difference: 5.6 min [95%CI 5.4 to 5.7; *P* < 0.001]). Travel time to opioid treatment programs was significantly longer than to Federally Qualified Health Centers (difference: -7.4 min [95%CI − 7.6 to − 7.2; *P* < 0.001]), dialysis facilities (difference: -8.2 min [95%CI − 8.4 to − 8.1; *P* < 0.001]), and hospitals (difference: − 2.8 min [95%CI − 3.0 to − 2.6; *P* < 0.001]). Compared with NYC, mean travel time to each type of SUD treatment program was significantly shorter in urban non-NYC areas and longer in rural areas. For robustness, compared with NYC, there was no significant difference in urban non-NYC areas for outpatient and residential SUD treatment programs, but more limited robustness for opioid treatment programs in urban non-NYC areas and all types of SUD treatment programs in rural areas.

**Conclusion:**

We identified widespread spatial accessibility of SUD treatment facilities across NYS. Recent opportunities such as revised federal regulations on opioid treatment program mobile medication units, increased flexibility in using telehealth in opioid treatment programs and other settings, and opioid settlement funding can be leveraged to increase access in rural areas.

## Introduction

In 2023, an estimated 17.1% of the Americans age 12 years or older—48.5 million people—had a substance use disorder (SUD) in the prior year [1]. New York State (NYS) has similar findings, with an estimated 17.1% of individuals age 12 or older having an SUD in the prior year [2]. Despite recent reductions in drug overdose deaths in both the US broadly and in NYS, drug overdose remains a major public health issue [3, 4]. Improving access to SUD treatment is an important intervention to reduce SUD-related morbidity and mortality including fatal overdose [5–7].

Spatial accessibility of treatment is defined as the distance and travel time to a treatment program [8, 9]. Spatial accessibility is a critical component of access and, for SUD treatment programs, it is associated with treatment outcomes through several pathways. First, longer travel times are consistently identified as a barrier to initiation of SUD treatment, with patients valuing proximity and convenience [10–14]. Second, longer travel times are associated with reduced continuity of treatment, including missed visits, lower retention, and shorter duration of treatment [15–21]. Third, longer travel times are associated with lower receipt of follow-up treatment [22–25]. Finally, the impact of spatial accessibility is magnified for some programs such as opioid treatment programs (OTP), which can require up to daily visits [26].

Estimating spatial accessibility through estimation of travel times can be a useful tool for understanding the treatment landscape; however, it is important to account for the local context. For example, studies calculating drive time may not reliably estimate travel times in areas where people mainly use public transit [27]. Further, estimation of travel time to a single nearest facility may underestimate treatment gaps due to barriers to access at that facility, such as the facility not accepting new patients, insurance participation, or specific treatment services offered [28]. Therefore, estimates of the robustness of spatial accessibility are also needed.

With new opportunities to expand SUD treatment through regulatory changes and opioid settlement funding, an analysis of spatial accessibility of SUD treatment can inform focused policy interventions to improve access. Therefore, the purpose of the current study is to examine the spatial accessibility of SUD treatment in NYS, benchmarked against other health care facilities. Because of known disparities in SUD treatment access and outcomes between urban and rural areas (i.e., urbanicity) broadly and in NYS [28–32], we analyzed spatial accessibility by urbanicity. Further, as spatial accessibility can vary regionally within a state, we also analyzed spatial accessibility by NYS economic development region, which is a regional classification maintained by NYS government [33].

## Methods

We performed a cross-sectional analysis of spatial accessibility SUD treatment programs and compared with other health care facilities in New York State. Similar to a previous analysis, comparison facilities included dialysis facilities, Federally Qualified Health Centers (FQHC), and hospitals [34]. We selected these facilities as comparators based on previously published research and potential similarities in frequency of attendance, as well as a requirement for licensure, specialized equipment, or both [35, 36]. The purpose of comparison facilities was to help contextualize the spatial accessibility of SUD treatment programs by providing points of reference from other health care facilities.

We obtained facility data in March 2024. First, we extracted addresses for all outpatient SUD treatment programs, OTPs, and residential SUD treatment programs from the NYS Office of Addiction Services and Supports provider and program search tool [37, 38]. Next, for dialysis facilities, we obtained addresses from the Centers for Medicare & Medicaid Services (CMS) [39]. Next, for FQHCs and acute care hospitals, we obtained geocoded location data (geocoordinates) from the Health Resources and Services Administration (HRSA) [40]. Because the focus of this study was on NYS, we did not include facilities in surrounding states.

For SUD treatment programs and dialysis facilities, we geocoded addresses with ArcGIS Pro version 3.2.2 (Esri, 2024) using a three-step procedure [41]. First, we standardized street addresses to include street, city, state, and five-digit ZIP code. Second, we geocoded street addresses, matching street addresses to the StreetMap Premium (Esri, 2024) database giving geographic coordinates. Every possible match is scored (0 to 100) indicating the degree of similarity between the street address and an address within the database. Third, we reviewed geocoding results, including individual review (using Google Maps) if an address was matched to a non-specific location (e.g., the geographic center of a ZIP code), was tied in match score with another location, or had a low match score (i.e., less than 80). We assigned final geographic coordinates upon confirmation of the correct location.

For NYS areas outside of New York City (NYC), we estimated travel time using drive time, based on data from StreetMap Premium. For NYC, we estimated travel time using public transit, based on a transit network dataset that modeled walking and using public transit (bus, subway, rail) [34]. To create this dataset, first we obtained publicly available transit schedules (General Transit Feed Specification files) from the Metropolitan Transportation Authority [42] and streets data from the Census Bureau [43]. Next, we used ArcGIS to connect transit stops to streets and create the transit network, allowing for walking and transfers between transit stops. Finally, we hand reviewed the transit network to identify and remove problematic features such as small streets that were disconnected from the greater street network (e.g., in parks). These features could artificially limit access to public transit, whereas a real pedestrian would use a footpath or other walkway that was not in our streets data.

We focused on two measures of spatial accessibility: one-way travel time and robustness. We estimated all travel times at the block group level [44]. We obtained geocoordinates of the centers of population of block groups calculated by the Census Bureau (year 2020) and removed all block groups with a zero population [44]. For robustness, we calculated the difference between the travel time to the closest and the fifth closest facility. We selected the fifth closest facility based on recent research and to represent a meaningful range of facilities [28]. We estimated all travel times using the ArcGIS nearest facility locator, modeling an 8:00 am departure on a weekday. [45]

**Fig. 1:**
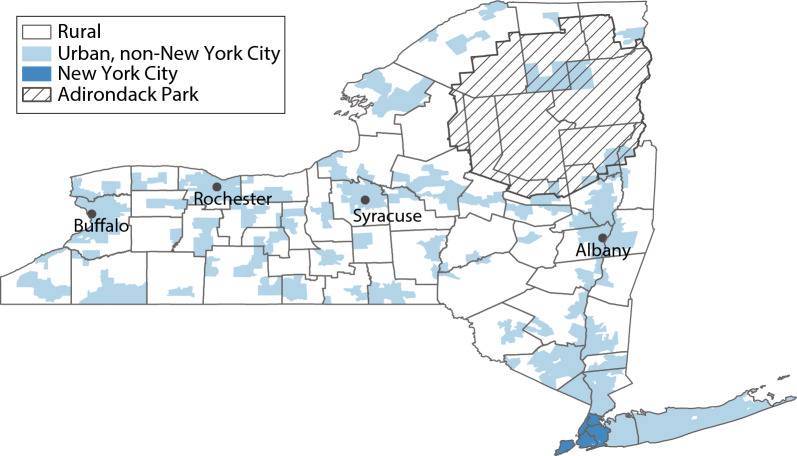
New York City, urban non-New York City, and rural areas in New York State. Urbanicity is displayed at the level of the block group. New York City is defined by its 5 constituent boroughs (counties). Outside of New York City, classification of block groups as urban is defined by containing at least one urban block, as defined by the Census Bureau. The shaded area indicates Adirondack Park, an area of limited human habitation. Grey borders represent counties. *SUD* substance use disorder

For urban and rural classification, we obtained urbanicity data from the Census bureau for each block [46], and counted a block group as urban if it contained at least one urban block (Fig. 1). We evaluated other indicators of urban and rural status, but these were typically at the county level, which was not sufficiently granular for this analysis. For NYS economic development regions, we obtained the list of counties in each region from the NYS Department of Health [33]. In all maps, we also depict the area of Adirondack Park, a major park in northern NYS which is a mixture of forest preserve and limited human habitation [47].

### Statistical analysis

First, we performed descriptive analyses of estimated travel times and robustness overall, by urbanicity, and by economic development region. We analyzed travel time as both a continuous variable and at different thresholds (≤ 15, ≤ 30, ≤ 45, and ≤ 60 min). We analyzed robustness only as a continuous variable. Because block groups have significantly differing populations, we calculated estimated travel times to represent the full statewide population by weighting all analyses by the 2022 5-year American Community Survey block group population estimates [48]. Next, using weighted paired t-tests, we compared travel times and robustness between different facilities overall, by urbanicity, and by economic development region. We performed all statistical analyses with SAS 9.4 (SAS Institute Inc., 2023). A two-tailed P-value of ≤ 0.05 was considered statistically significant.

## Results

In NYS, we identified 15,623 block groups with a total population of 19,994,326. There were 516 outpatient SUD treatment programs, 110 opioid treatment programs, 186 residential SUD treatment programs, 534 Federally Qualified Health Centers, 347 dialysis facilities, and 135 hospitals in NYS.

**Fig. 2:**
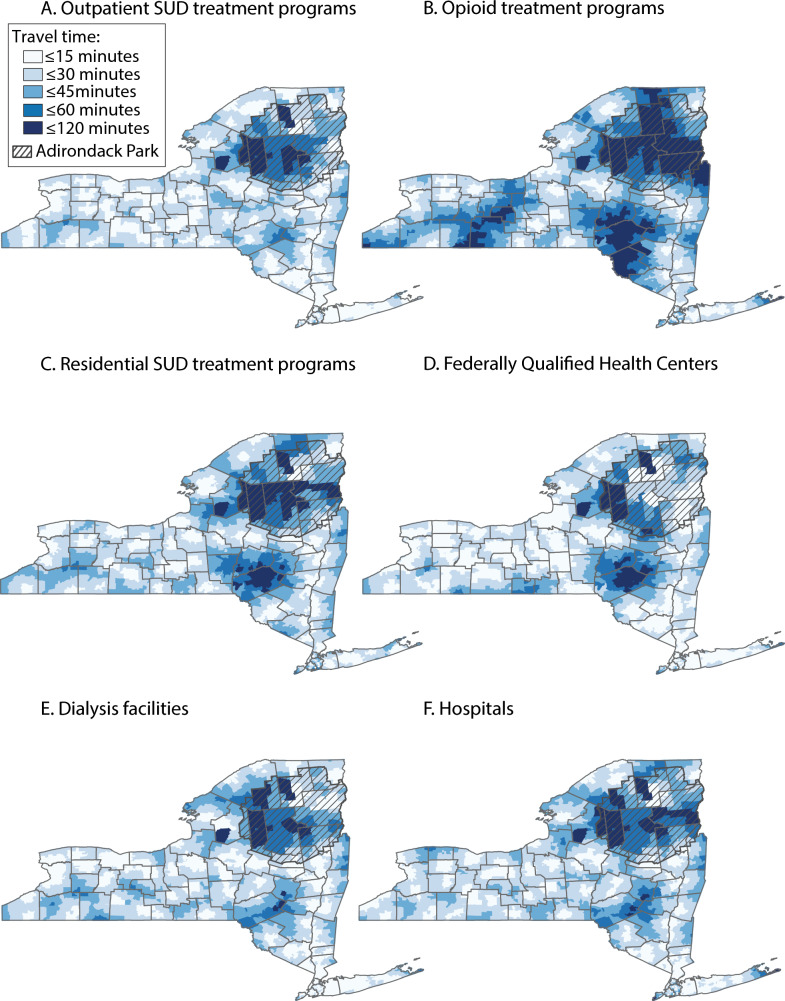
Areas within one-way travel time thresholds to substance use disorder treatment programs and other health care facilities, by county, New York State, 2024. Maps show travel times at the level of the block group. The shaded area indicates Adirondack Park, an area of limited human habitation. Grey borders represent counties. *SUD* substance use disorder

Statewide, the percentage of NYS residents within 30 min travel time of an SUD treatment program was 97.2% for outpatient SUD treatment programs, 82.3% for opioid treatment programs, and 86.7% for residential SUD treatment programs (Table 1, Fig. 2). The mean travel time was 11.1 min (SD: 7.6) to outpatient SUD treatment programs, 19.5 min (SD: 12.8) to OTPs, and 17.9 min (SD: 11.1) to residential SUD treatment programs.

**Table 1:** One-way travel time to substance use disorder treatment programs and other health care facilities, by urbanicity, New York State, 2024

Urbanicity^a^	Outpatient SUD treatment programs	Opioid treatment programs	Residential SUD treatment programs	Federally Qualified Health Centers	Dialysis facilities	Hospitals
Statewide^b^						
Minutes, mean (SD)	11.1 (7.6)	19.5 (12.8)	17.9 (11.1)	12.1 (9.1)	11.2 (7.4)	16.7 (9.9)
Minutes, median (IQR)	9.2 (5.5, 14.9)	16.2 (10.5, 25.2)	15.8 (9.9, 23.8)	10.0 (5.9, 15.3)	9.5 (6.1, 14.5)	14.8 (9.5, 21.8)
≤ 15 min, %	75.4	44.8	45.5	74.1	77.0	51.1
≤ 30 min, %	97.2	82.3	86.7	94.9	97.1	90.7
≤ 45 min, %	99.8	95.2	97.6	98.9	99.7	98.5
≤ 60 min, %	100	98.7	99.7	99.8	99.9	99.6
New York City						
Minutes, mean (SD)	12.6 (7.3)	20.4 (10.9)	21.4 (11.1)	12.2 (8.7)	11.9 (5.8)	20.2 (9.9)
Minutes, median (IQR)	11.2 (7.3, 16.6)	18.7 (11.9, 27.6)	19.5 (13.3, 28.0)	10.2 (6.0, 15.6)	11.3 (7.7, 15.1)	19.0 (13.5, 25.4)
≤ 15 min, %	69.2	38.2	31.0	72.5	74.8	32.1
≤ 30 min, %	97.0	79.7	79.9	95.1	99.0	85.9
≤ 45 min, %	100	98.0	96.7	99.2	100	97.8
≤ 60 min, %	100	100	99.9	99.9	100	99.3
Urban, non-New York City						
Minutes, mean (SD)	7.9 (5.1)	15.7 (11.2)	13.2 (8.2)	10.2 (7.2)	8.4 (5.5)	11.8 (6.8)
Minutes, median (IQR)	6.6 (4.2, 10.1)	13.3 (8.6, 19.0)	12.1 (7.1, 17.2)	8.8 (5.4, 12.8)	7.1 (4.8, 10.3)	10.9 (7.1, 15.0)
≤ 15 min, %	90.1	57.9	64.6	83.0	89.8	75.1
≤ 30 min, %	99.7	91.4	96.3	97.8	99.1	98.5
≤ 45 min, %	100	96.2	99.4	99.4	100	99.6
≤ 60 min, %	100	99.0	99.9	100	100	99.9
Rural						
Minutes, mean (SD)	20.8 (9.3)	34.9 (16.7)	26.7 (12.7)	21.5 (12.9)	23.1 (10.1)	25.3 (10.8)
Minutes, median (IQR)	19.2 (14.5, 25.9)	31.7 (22.3, 43.4)	24.5 (18.2, 33.0)	18.5 (12.8, 27.7)	21.4 (16.0, 28.8)	23.7 (17.9, 31.1)
≤ 15 min, %	27.4	6.6	14.4	35.0	20.7	15.0
≤ 30 min, %	84.6	46.3	68.2	79.1	77.8	72.3
≤ 45 min, %	98.5	77.2	92.3	94.2	97.1	95.4
≤ 60 min, %	99.6	90.9	97.6	98.5	99.5	98.9

^a^Urban areas outside of New York City are defined at the block group level where at least one constituent block is classified as urban by the Census Bureau

^b^Includes New York City, urban non-New York City, and rural areas

*IQR* interquartile range, *SD* standard deviation, *SUD* substance use disorder

Statewide mean travel time to outpatient SUD treatment programs was comparable to that for FQHCs (1.0 min longer to FQHCs [95% CI 0.9 to 1.1; *P* < 0.001]) and dialysis facilities (0.1 min longer to dialysis facilities [95% CI 0.03 to 0.2; *P* = 0.01]), and significantly shorter than to hospitals (5.6 min longer to hospitals [95% CI 5.4 to 5.7; *P* < 0.001]; Table 2). Travel time to OTPs was significantly longer than to FQHCs (− 7.4 min shorter to FQHCs [95% CI − 7.6 to − 7.2; *P* < 0.001]), dialysis facilities (− 8.2 min shorter to dialysis facilities [95% CI − 8.4 to − 8.1; *P* < 0.001]), and hospitals (− 2.8 min shorter to hospitals [95% CI − 3.0 to − 2.6; *P* < 0.001]). Mean statewide travel time to residential SUD treatment programs was also significantly longer than to FQHCs (−5.9 min shorter to FQHCs [95% CI − 6.0 to − 5.7; *P* < 0.001]), dialysis facilities (− 6.7 min shorter to dialysis facilities [95% CI − 6.8 to − 6.5; *P* < 0.001]), and hospitals (− 1.3 min shorter to hospitals [95% CI − 1.4 to − 1.1; *P* < 0.001]). Compared with NYC, mean travel time to each type of SUD treatment program was significantly shorter in urban, non-NYC areas and longer in rural areas (Table 3).

**Table 2:** Comparison of one-way travel time to substance use disorder treatment programs and other health care facilities, by urbanicity, New York State, 2024

	Comparison facility		
	Federally qualified health centers	Dialysis facilities	Hospitals
Urbanicity^a^	Difference, minutes (95% CI)^b^		
Statewide^c^			
Outpatient SUD treatment programs	1.0 (0.9, 1.1)***	0.1 (0.03, 0.2)*	5.6 (5.4, 5.7)***
Opioid treatment programs	−7.4 (−7.6, −7.2)***	−8.2 (−8.4, −8.1)***	−2.8 (−3.0, −2.6)***
Residential SUD treatment programs	−5.9 (−6.0, −5.7)***	−6.7 (−6.8, −6.5)***	−1.3 (−1.4, −1.1)***
New York City			
Outpatient SUD treatment programs	−0.5 (−0.6, −0.3)***	−0.8 (−0.9, −0.6)***	7.6 (7.4, 7.8)***
Opioid treatment programs	−8.2 (−8.4, −8.0)***	−8.5 (−8.7, −8.2)***	−0.1 (−0.4, 0.1)
Residential SUD treatment programs	−9.2 (−9.4, −9.0)***	−9.5 (−9.7, −9.2)***	−1.1 (−1.4, −0.9)***
Urban, non−New York City			
Outpatient SUD treatment programs	2.3 (2.2, 2.5)***	0.5 (0.4, 0.7)***	3.9 (3.8, 4.1)***
Opioid treatment programs	−5.5 (−5.8, −5.3)***	−7.3 (−7.6, −7.1)***	−4.0 (−4.2, −3.7)***
Residential SUD treatment programs	−3.0 (−3.2, −2.8)***	−4.8 (−4.9, −4.6)***	−1.4 (−1.6, −1.2)***
Rural			
Outpatient SUD treatment programs	0.7 (0.1, 1.2)*	2.2 (1.8, 2.7)***	4.5 (4.0, 4.9)***
Opioid treatment programs	−13.4 (−14.2, −12.6)***	−11.8 (−12.5, −11.1)***	−9.6 (−10.4, −8.8)***
Residential SUD treatment programs	−5.3 (−5.8, −4.7)***	−3.7 (−4.2, −3.2)***	−1.5 (−2.0, −0.9)***

^a^Urban areas outside of New York City are defined at the block group level where at least one constituent block is classified as urban by the Census Bureau

^b^A positive number for a given facility type (row label) indicates that the mean travel time to the comparison facility type (column label) is longer than to the given facility type. A negative number indicates that the mean travel time to the comparison facility type is shorter than to the given facility type. Comparisons are made within urbanicity categories

^c^Includes New York City, urban non-New York City, and rural areas

^*^P ≤ 0.05

^**^P ≤ 0.01

^***^P < 0.001

*95% CI* 95% confidence interval, *SUD* substance use disorder

**Table 3:** Mean difference in one-way travel time to substance use disorder treatment programs, by urbanicity, New York State, 2024

		Outpatient SUD treatment programs		Opioid treatment programs		Residential SUD treatment programs	
Urbanicity^a^	Population (%)	Difference, minutes (95% CI)^b^	*P*	Difference, minutes (95% CI)^b^	*P*	Difference, minutes (95% CI)^b^	*P*
New York City	8,622,467 (43.1)	Ref		Ref		Ref	
Urban, non-New York City	9,555,757 (47.8)	−4.8 (−5.0, −4.6)	< 0.001	−4.6 (−5.0, −4.2)	< 0.001	−8.2 (−8.5, −7.9)	< 0.001
Rural	1,816,102 (9.1)	8.2 (7.7, 8.7)	< 0.001	14.5 (13.7, 15.4)	< 0.001	5.4 (4.7, 6.1)	< 0.001

^a^Urban areas outside of New York City are defined at the block group level where at least one constituent block is classified as urban by the Census Bureau

^b^A positive number for a given urbanicity indicates that the mean travel time to that facility type is longer, compared with New York City. A negative number indicates that the mean travel time is shorter, compared with New York City

*SUD* substance use disorder

Statewide, the difference in travel time between the closest and fifth closest facility (i.e., robustness) for outpatient SUD treatment programs was comparable to the difference for FQHCs (0.4 min longer for FQHCs [95% CI 0.3 to 0.5; *P* < 0.001]) and significantly shorter than dialysis facilities (3.9 min longer for dialysis facilities [95% CI 3.8 to 4.1; *P* < 0.001]) and hospitals (10.9 min longer for hospitals [95% CI 10.7 to 11.1; *P* < 0.001]; Table 4). For OTPs, the difference between the closest and fifth closest facility was significantly longer than for FQHCs (−12.3 min shorter for FQHCs [95% CI − 12.5 to − 12.0; *P* < 0.001]), dialysis facilities (− 8.7 min shorter for dialysis facilities [95% CI − 9.0 to − 8.5; *P* < 0.001]), and hospitals (− 1.8 min shorter for hospitals [95% CI − 2.0 to − 1.5; *P* < 0.001]). For residential SUD treatment programs, the difference between the closest and fifth closes facility was significantly longer than for FQHCs (− 5.4 min shorter for FQHCs [95% CI − 5.6 to − 5.2]; *P* < 0.001), dialysis facilities (− 1.9 min shorter for dialysis facilities [95% CI − 2.1 to − 1.7; *P* < 0.001]), and significantly shorter than hospitals (5.0 min longer for hospitals [95% CI 4.9 to 5.2; *P* < 0.001]). Compared with NYC, the difference between the closest and fifth closest facility was not significantly different in urban, non-NYC areas for outpatient SUD treatment programs and residential SUD treatment programs, but significantly longer for opioid treatment programs in non-NYC areas and all types of programs in rural areas (Table 5).

**Table 4:** Comparisons of differences in one-way travel time between the closest and fifth closest health care facility, by urbanicity, New York State, 2024

	Comparison facility		
	Federally Qualified Health Centers	Dialysis facilities	Hospitals
Urbanicity^a^	Difference, minutes (95% CI)^b^		
Statewide^c^			
Outpatient SUD treatment programs	0.4 (0.3, 0.5)***	3.9 (3.8, 4.1)***	10.9 (10.7, 11.1)***
Opioid treatment programs	−12.3 (−12.5, −12.0)***	−8.7 (−9.0, −8.5)***	−1.8 (−2.0, −1.5)***
Residential SUD treatment programs	−5.4 (−5.6, −5.2)***	−1.9 (−2.1, −1.7)***	5.0 (4.9, 5.2)***
New York City			
Outpatient SUD treatment programs	−1.3 (−1.5, −1.1)***	1.9 (1.7, 2.1)***	8.6 (8.4, 8.8)***
Opioid treatment programs	−6.7 (−7.0, −6.4)***	−3.5 (−3.8, −3.3)***	3.2 (2.9, 3.4)***
Residential SUD treatment programs	−7.0 (−7.3, −6.7)***	−3.8 (−4.1, −3.6)***	2.9 (2.6, 3.1)***
Urban, non−New York City			
Outpatient SUD treatment programs	1.8 (1.6, 2.0)***	4.7 (4.4, 4.9)***	11.9 (11.7, 12.2)***
Opioid treatment programs	−15.6 (−16.0, −15.1)***	−12.7 (−13.1, −12.4)***	−5.5 (−5.9, −5.1)***
Residential SUD treatment programs	−4.1 (−4.3, −3.8)***	−1.2 (−1.4, −1.0)***	6.0 (5.8, 6.3)***
Rural			
Outpatient SUD treatment programs	1.1 (0.5, 1.8)***	9.5 (8.7, 10.3)***	16.3 (15.4, 17.1)***
Opioid treatment programs	−21.0 (−22.2, −19.7)***	−12.6 (−13.6, −11.5)***	−5.8 (−6.9. −4.7)***
Residential SUD treatment programs	−5.0 (−5.9, −4.2)***	3.4 (2.7, 4.0)***	10.1 (9.4, 10.8)***

^a^Urban areas outside of New York City are defined at the block group level where at least one constituent block is classified as urban by the Census Bureau

^b^A positive number for a given facility type (row label) indicates that the robustness (i.e., the mean difference in travel time between the closest and fifth closest facility) is better than for the comparison facility type (column label). A negative number indicates that the robustness of the given facility type is worse than for the comparison facility type. Comparisons are made within urbanicity categories

^c^Includes New York City, urban non-New York City, and rural areas

^*^P ≤ 0.05

^**^P ≤ 0.01

^***^P < 0.001

*95% CI* 95% confidence interval, *SUD* substance use disorder

**Table 5:** Comparisons of differences in one-way travel time between the closest and fifth closest substance use disorder treatment program, by urbanicity, New York State, 2024

		Outpatient SUD treatment programs		Opioid treatment programs		Residential SUD treatment programs	
Urbanicity^a^	Population (%)	Difference, minutes (95% CI)^b^	*P*	Difference, minutes (95% CI)^b^	*P*	Difference, minutes (95% CI)^b^	*P*
New York City	8,622,467 (43.1)	Ref		Ref		Ref	
Urban, non-New York City	9,555,757 (47.8)	−0.06 (−0.3, 0.2)	0.66	11.9 (11.3, 12.5)	< 0.001	0.08 (−0.3, 0.5)	0.71
Rural	1,816,102 (9.1)	7.0 (6.4, 7.6)	< 0.001	23.7 (22.2, 25.2)	< 0.001	7.5 (6.5, 8.5)	< 0.001

^a^Urban areas outside of New York City are defined at the block group level where at least one constituent block is classified as urban by the Census Bureau

^b^A positive number for a given urbanicity indicates that the robustness (i.e., the mean difference in travel time between the closest and fifth closest facility) for that facility type is worse, compared with New York City. A negative number indicates that the robustness is better, compared with New York City

*95% CI* 95% confidence interval, *SUD* substance use disorder

**Fig. 3:**
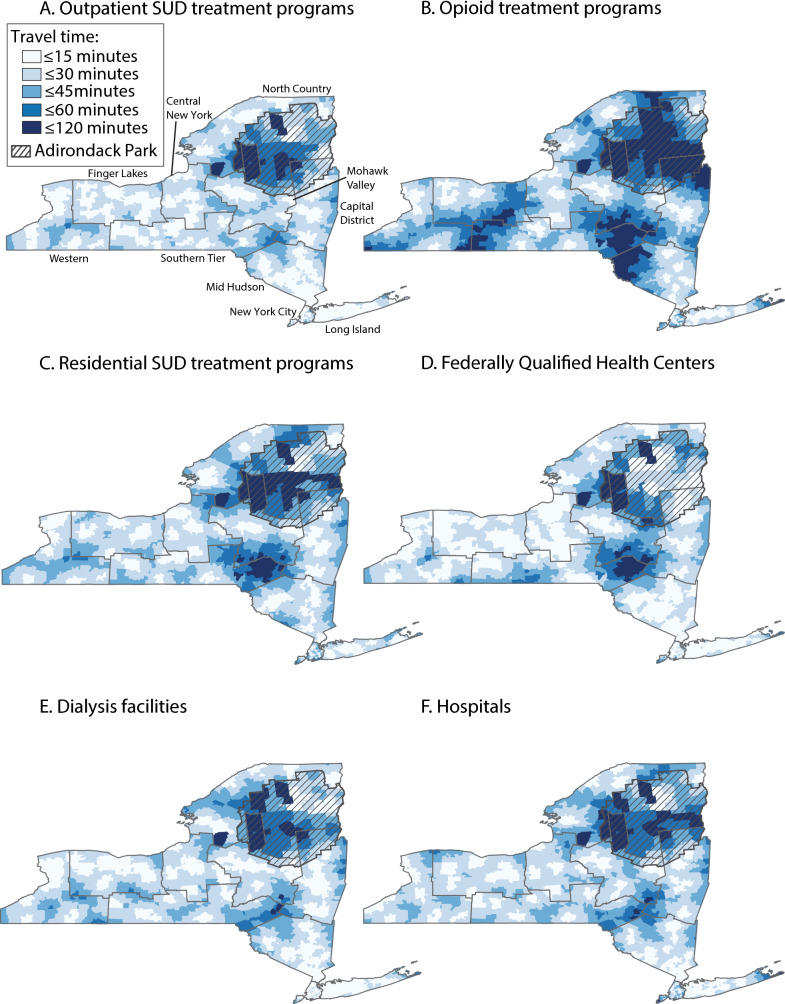
Areas within one-way travel time thresholds to substance use disorder treatment programs and other health care facilities, by New York State economic development region, 2024. Maps show travel times at the level of the block group. The shaded area indicates Adirondack Park, an area of limited human habitation. Grey borders represent economic development region borders. *SUD* substance use disorder

By NYC economic development region (Tables 6, 7, 8, 9, 10, Fig. 3) and compared with New York City, the mean travel time to outpatient SUD treatment programs was significantly shorter in 5 economic development regions (Capital District, Finger Lakes, Long Island, Mid Hudson, and Western), significantly longer in 2 economic development regions (Mohawk Valley and North Country), and not significantly different in 2 economic development regions (Central New York and Southern Tier). For OTPs, compared with New York City, the mean travel time was significantly shorter in 4 economic development regions (Central New York, Long Island, Mid Hudson, and Western) and significantly longer in 5 economic development regions (Capital District, Finger Lakes, Mohawk Valley, North Country, and Southern Tier). For residential SUD treatment programs, compared with New York City, the mean travel time was significantly shorter in 6 economic development regions (Capital District, Central New York, Finger Lakes, Long Island, Mid Hudson, and Western), significantly longer in 1 economic development region (North Country), and not significantly different in 2 economic development regions (Mohawk Valley and Southern Tier).

**Table 6:** One-way travel time to substance use disorder treatment programs and other health care facilities, by economic development region, New York State, 2024

Economic development region	Outpatient SUD treatment programs	Opioid treatment programs	Residential SUD treatment programs	Federally qualified health centers	Dialysis facilities	Hospitals
New York City						
Minutes, mean (SD)	12.6 (7.3)	20.4 (10.9)	21.4 (11.1)	12.2 (8.7)	11.9 (5.8)	20.2 (9.9)
Minutes, median (IQR)	11.2 (7.3, 16.6)	18.7 (11.9, 27.6)	19.5 (13.3, 28.0)	10.2 (6.0, 15.6)	11.3 (7.7, 15.1)	19.0 (13.5, 25.4)
≤ 15 min, %	69.2	38.2	31.0	72.4	74.8	32.1
≤ 30 min, %	97.0	79.7	79.9	95.1	99.0	85.9
≤ 45 min, %	100	98.0	96.7	99.2	100	97.8
≤ 60 min, %	100	100	99.9	99.9	100	99.3
Capital District						
Minutes, mean (SD)	12.0 (8.7)	23.4 (18.9)	13.2 (9.2)	13.7 (9.0)	11.8 (8.9)	15.6 (9.5)
Minutes, median (IQR)	9.8 (5.6, 23.0)	17.0 (8.7, 34.9)	11.4 (6.4, 17.5)	11.7 (6.6, 19.4)	9.5 (6.2, 14.4)	13.9 (9.2, 20.1)
≤ 15 min, %	70.6	46.4	65.6	61.6	77.1	56.5
≤ 30 min, %	95.2	69.2	93.9	94.4	94.3	92.3
≤ 45 min, %	99.5	85.4	99.3	99.7	99.1	98.8
≤ 60 min, %	99.9	94.8	99.9	100	99.9	99.9
Central New York						
Minutes, mean (SD)	12.5 (8.2)	16.2 (10.6)	12.3 (8.4)	10.3 (6.3)	11.0 (7.6)	14.4 (8.3)
Minutes, median (IQR)	10.7 (5.6, 17.6)	14.1 (8.2, 21.5)	11.0 (5.8, 16.5)	9.7 (5.4, 14.1)	9.0 (5.3, 14.7)	13.4 (7.6, 19.6)
≤ 15 min, %	65.8	53.7	69.6	79.2	75.7	56.4
≤ 30 min, %	96.0	88.2	95.4	99.0	97.6	95.5
≤ 45 min, %	100	98.9	99.7	100	100	99.9
≤ 60 min, %	100	100	100	100	100	100
Finger Lakes						
Minutes, mean (SD)	11.0 (6.8)	22.7 (13.9)	13.9 (9.1)	11.5 (6.3)	11.1 (7.8)	14.5 (8.1)
Minutes, median (IQR)	9.9 (5.3, 15.6)	18.8 (13.2, 28.8)	12.6 (5.8, 19.6)	11.7 (5.9, 16.2)	8.8 (5.7, 14.2)	13.6 (8.0, 20.0)
≤ 15 min, %	72.3	32.7	59.3	68.2	77.1	56.7
≤ 30 min, %	99.2	76.3	94.5	99.8	96.0	96.1
≤ 45 min, %	100	89.1	99.9	100	99.8	99.8
≤ 60 min, %	100	98.6	100	100	100	100
Long Island						
Minutes, mean (SD)	6.6 (4.1)	13.9 (7.2)	14.1 (6.5)	9.2 (4.8)	7.2 (4.8)	11.3 (6.6)
Minutes, median (IQR)	5.8 (4.0, 8.1)	13.0 (9.5, 16.9)	13.8 (9.4, 17.7)	8.7 (6.1, 11.3)	6.2 (4.4, 8.5)	10.7 (7.7, 13.6)
≤ 15 min, %	96.2	64.1	58.0	91.9	94.2	83.2
≤ 30 min, %	99.6	98.0	98.2	99.3	99.0	98.3
≤ 45 min, %	100	99.0	100	99.9	99.9	99.0
≤ 60 min, %	100	99.8	100	100	100	99.8
Mid Hudson						
Minutes, mean (SD)	9.2 (6.1)	18.5 (12.4)	16.9 (9.4)	9.3 (5.7)	11.1 (7.1)	12.9 (7.4)
Minutes, median (IQR)	7.7 (4.8, 11.8)	16.6 (9.8, 22.2)	15.5 (9.9, 22.7)	8.4 (4.8, 12.3)	9.4 (6.0, 14.5)	12.0 (7.5, 16.4)
≤ 15 min, %	85.6	42.1	47.3	85.4	76.7	69.4
≤ 30 min, %	98.6	86.6	89.5	99.5	97.4	96.8
≤ 45 min, %	100	95.6	99.6	100	100	99.9
≤ 60 min, %	100	98.4	100	100	100	100
Mohawk Valley						
Minutes, mean (SD)	14.1 (9.9)	22.8 (18.4)	20.3 (17.1)	23.9 (17.8)	13.3 (10.5)	16.0 (11.6)
Minutes, median (IQR)	11.4 (6.5, 20.6)	18.0 (8.1, 33.0)	15.0 (7.4, 29.0)	18.8 (8.4, 39.2)	9.6 (5.9, 18.0)	12.1 (7.3, 22.5)
≤ 15 min, %	59.6	43.3	49.6	44.5	67.4	58.8
≤ 30 min, %	93.4	71.7	76.1	64.1	91.3	85.9
≤ 45 min, %	99.6	85.9	88.7	83.9	99.4	98.7
≤ 60 min, %	99.7	92.2	96.2	98.2	99.6	99.6
North Country						
Minutes, mean (SD)	17.2 (13.2)	30.8 (20.9)	23.7 (16.6)	20.3 (12.9)	20.8 (13.5)	24.3 (16.7)
Minutes, median (IQR)	15.8 (6.4, 24.3)	26.5 (15.3, 41.0)	21.1 (10.9, 32.5)	19.0 (9.4, 28.0)	19.6 (10.0, 28.2)	21.6 (11.5, 34.1)
≤ 15 min, %	48.3	23.7	34.9	37.3	38.2	33.2
≤ 30 min, %	84.5	57.0	71.6	80.6	78.3	68.5
≤ 45 min, %	96.5	78.4	87.5	95.4	95.5	87.5
≤ 60 min, %	98.8	87.1	96.3	99.3	98.9	96.4
Southern Tier						
Minutes, mean (SD)	13.1 (8.4)	25.6 (19.5)	20.7 (16.1)	23.5 (15.7)	16.4 (10.9)	17.3 (10.6)
Minutes, median (IQR)	11.5 (6.7, 17.8)	19.2 (10.4, 36.1)	16.0 (9.0, 26.7)	19.0 (11.6, 34.6)	14.3 (7.8, 23.2)	15.2 (9.1, 22.7)
≤ 15 min, %	65.3	38.9	47.5	37.5	54.5	48.8
≤ 30 min, %	95.7	68.4	80.2	68.6	88.0	87.0
≤ 45 min, %	99.3	81.0	90.3	91.1	98.0	98.0
≤ 60 min, %	100	93.7	96.4	97.1	99.7	99.6
Western						
Minutes, mean (SD)	9.2 (7.5)	16.1 (12.0)	13.1 (9.1)	10.8 (7.2)	9.8 (8.3)	13.6 (8.4)
Minutes, median (IQR)	6.5 (4.1, 11.9)	12.0 (8.3, 20.4)	11.2 (6.2, 16.9)	9.7 (5.3, 14.5)	6.9 (4.8, 11.0)	11.6 (6.8, 18.7)
≤ 15 min, %	82.8	62.9	65.6	77.8	81.5	61.8
≤ 30 min, %	97.1	87.5	93.6	97.8	95.8	95.3
≤ 45 min, %	99.9	96.0	99.6	99.8	99.6	99.9
≤ 60 min, %	100	99.7	100	100	100	100

*IQR* interquartile range, *SD* standard deviation, *SUD* substance use disorder

**Table 7:** Comparison of one-way travel time between substance use disorder treatment programs and other health care facilities, by economic development region, New York State, 2024

	Comparison facility		
	Federally Qualified Health Centers	Dialysis facilities	Hospitals
Economic development region	Difference, minutes (95% CI)^a^		
New York City			
Outpatient SUD treatment programs	−0.5 (−0.6, −0.3)***	−0.8 (−0.9, −0.6)***	7.6 (7.4, 7.8)***
Opioid treatment programs	−8.2 (−8.4, −8.0)***	−8.5 (−8.7, −8.2)***	−0.1 (−0.4, 0.1)
Residential SUD treatment programs	−9.2 (−9.4, −9.0)***	−9.5 (−9.7, −9.2)***	−1.1 (−1.4, −0.9)***
Capital District			
Outpatient SUD treatment programs	1.7 (1.3, 2.1)***	−0.2 (−0.5, 0.1)	3.6 (3.3, 3.9)***
Opioid treatment programs	−9.7 (−10.8, −8.6)***	−11.6 (−12.5, −10.6)***	−7.8 (−8.8, −6.7)***
Residential SUD treatment programs	0.5 (0.1, 0.9)**	−1.3 (−1.7, −1.0)***	2.5 (2.1, 2.8)***
Central New York			
Outpatient SUD treatment programs	−2.2 (−2.7, −1.7)***	−1.4 (−2.0, −0.9)***	1.9 (1.5, 2.4)***
Opioid treatment programs	−5.9 (−6.6, −5.1)***	−5.1 (−5.9, −4.4)***	−1.8 (−2.5, −1.0)***
Residential SUD treatment programs	−2.0 (−2.6, −1.5)***	−1.3 (−1.7, −0.9)***	2.1, (1.8, 2.4)***
Finger Lakes			
Outpatient SUD treatment programs	0.5 (0.1, 0.9)**	0.1 (−0.3, 0.5)	3.5 (3.2, 3.9)***
Opioid treatment programs	−11.2 (−12.1, −10.4)***	−11.6 (−12.3, −10.9)***	−8.2 (−9.0, −7.3)***
Residential SUD treatment programs	−2.4 (−2.9, −1.8)***	−2.8 (−3.2, −2.3)***	0.7 (0.3, 1.0)***
Long Island			
Outpatient SUD treatment programs	2.6 (2.4, 2.8)***	0.6 (0.5, 0.8)***	4.8 (4.5, 5.1)***
Opioid treatment programs	−4.7 (−5.0, −4.4)***	−6.7 (−6.9, −6.4)***	−2.5 (−2.8, −2.3)***
Residential SUD treatment programs	−5.0 (−5.2, −4.7)***	−7.0 (−7.2, −6.7)***	−2.8 (−3.2, −2.4)***
Mid Hudson			
Outpatient SUD treatment programs	0.1 (−0.2, 0.3)	1.9 (1.6, 2.1)***	3.8 (3.5, 4.0)***
Opioid treatment programs	−9.2 (−9.7, −8.7)***	−7.4 (−7.8, −7.0)***	−5.5 (−6.0, −5.0)***
Residential SUD treatment programs	−7.7 (−8.1, −7.3)***	−5.9 (−6.3, −5.5)***	−4.0 (−4.5, −3.6)***
Mohawk Valley			
Outpatient SUD treatment programs	9.8 (8.4, 11.1)***	−0.8 (−1.6, −0.1)*	1.9 (1.0, 2.8)***
Opioid treatment programs	1.1 (0.05, 2.1)*	−9.5 (−11.0, −8.1)***	−6.8 (−8.2, −5.3)***
Residential SUD treatment programs	3.6 (2.4, 4.8)***	−7.0 (−8.2, −5.7)***	−4.2 (−5.7, −2.8)***
North Country			
Outpatient SUD treatment programs	3.0 (2.0, 4.1)***	3.6 (2.3, 4.8)***	7.1 (5.8, 8.4)***
Opioid treatment programs	−10.6 (−12.9, −8.3)***	−10.1 (−12.1, −8.0)***	−6.5 (−8.0, −5.0)***
Residential SUD treatment programs	−3.4 (−5.2, −1.7)***	−2.9 (−4.4, −1.4)***	0.6 (−0.1, 1.4)
Southern Tier			
Outpatient SUD treatment programs	10.4 (9.3, 11.5)***	3.3 (2.8, 3.9)***	4.2 (3.7, 4.7)***
Opioid treatment programs	−2.1 (−3.6, −0.5)**	−9.2 (−10.4, −7.9)***	−8.3 (−9.6, −7.0)***
Residential SUD treatment programs	2.8 (1.8, 3.9)***	−4.2 (−5.3, −3.2)***	−3.4 (−4.4, −2.4)***
Western			
Outpatient SUD treatment programs	1.6 (1.2, 1.9)***	0.6 (0.3, 0.8)***	4.4 (4.0, 4.8)***
Opioid treatment programs	−5.2 (−5.8, −4.7)***	−6.2 (−6.7, −5.8)***	−2.4 (−3.0, −1.9)***
Residential SUD treatment programs	−2.3 (−2.7, −1.9)***	−3.3 (−3.7, −2.9)***	0.5 (0.04, 1.0)*

^a^A positive number for a given facility type (row label) indicates that the mean travel time to the comparison facility type (column label) is longer than to the given facility type. A negative number indicates that the mean travel time to the comparison facility type is shorter than to the given facility type. Comparisons are made within economic development regions

^*^ *P* ≤ 0.05; ** *P* ≤ 0.01; *** *P* < 0.001

95% CI = 95% confidence interval; SUD = substance use disorder

**Table 8:** Mean difference in one-way travel time to substance use disorder treatment programs, by economic development region, New York State, 2024

		Outpatient SUD treatment programs		Opioid treatment programs		Residential SUD treatment programs	
Economic development region	Population (%)	Difference, minutes (95% CI)^a^	*P*	Difference, minutes (95% CI)^a^	*P*	Difference, minutes (95% CI)^a^	*P*
New York City	8,622,467 (43.1)	Ref		Ref		Ref	
Capital District	1,108,289 (5.5)	−0.6 (−1.2, −0.02)	0.04	3.0 (1.8, 4.3)	< 0.001	−8.2 (−8.9, −7.5)	< 0.001
Central New York	781,620 (3.9)	−0.2 (−0.8, 0.5)	0.61	−4.2 (−5.1, −3.4)	< 0.001	−9.0 (−9.7, −8.3)	< 0.001
Finger Lakes	1,219,052 (6.1)	−1.6 (−2.1, −1.1)	< 0.001	2.3 (1.4, 3.3)	< 0.001	−7.5 (−8.2, −6.9)	< 0.001
Long Island	2,913,593 (14.6)	−6.1 (−6.3, −5.8)	< 0.001	−6.5 (−6.9, −6.1)	< 0.001	−7.2 (−7.6, −6.8)	< 0.001
Mid Hudson	2,391,754 (12.0)	−3.5 (−3.8, −3.1)	< 0.001	−1.9 (−2.5, −1.3)	< 0.001	−4.4 (−4.9, −3.9)	< 0.001
Mohawk Valley	483,900 (2.4)	1.5 (0.6, 2.4)	0.002	2.5 (0.7, 4.2)	0.006	−1.1 (−2.7, 0.6)	0.20
North Country	422,507 (2.1)	4.6 (3.3, 5.9)	< 0.001	10.5 (8.4, 12.5)	< 0.001	2.3 (0.7, 4.0)	0.006
Southern Tier	636,020 (3.2)	0.5 (−0.2, 1.1)	0.20	5.2 (3.7, 6.8)	< 0.001	−0.7 (−2.0, 0.6)	0.31
Western	1,415,124 (7.1)	−3.4 (−3.9, −3.0)	< 0.001	−4.3 (−5.0, −3.6)	< 0.001	−8.3 (−8.8, −7.7)	< 0.001

^a^A positive number for a given economic development region indicates that the mean travel time to that facility type is longer, compared with New York City. A negative number indicates that the mean travel time is shorter, compared with New York City

*95% CI* 95% confidence interval, *SUD* substance use disorder

**Table 9:** Comparisons of differences in one-way travel time between the closest and fifth closest health care facility, by economic development region, New York State, 2024

	Comparison facility		
	Federally Qualified Health Centers	Dialysis facilities	Hospitals
Economic development region	Difference, minutes (95% CI)^a^		
New York City			
Outpatient SUD treatment programs	−1.3 (−1.5, −1.1)***	1.9 (1.7, 2.1)***	8.6 (8.4, 8.8)***
Opioid treatment programs	−6.7 (−7.0, −6.4)***	−3.5 (−3.8, −3.3)***	3.2 (2.9, 3.4)***
Residential SUD treatment programs	−7.0 (−7.3, −6.7)***	−3.8 (−4.1, −3.6)***	2.9 (2.6, 3.1)***
Capital District			
Outpatient SUD treatment programs	0.3 (−0.2, 0.9)	5.7 (5.3, 6.1)***	14.2 (13.6, 14.7)***
Opioid treatment programs	−4.8 (−5.4, −4.3)***	0.5 (−0.3, 1.4)	9.0 (8.1, 9.9)***
Residential SUD treatment programs	−5.4 (−6.2, −4.5)***	0.01 (−0.5, 0.5)	8.5 (8.0, 8.9)***
Central New York			
Outpatient SUD treatment programs	0.08 (−0.6, 0.8)	4.1 (3.7, 4.6)***	20.1 (19.3, 21.0)***
Opioid treatment programs	−19.1 (−20.2, −17.9)***	−15.0 (−16.2, −13.8)***	1.0 (0.3, 1.7)**
Residential SUD treatment programs	−2.9 (−3.4, −2.3)***	1.2 (0.8, 1.6)***	17.2 (16.4, 18.0)***
Finger Lakes			
Outpatient SUD treatment programs	0.6 (0.2, 1.0)**	3.4 (3.0, 3.8)***	5.5 (5.0, 5.9)***
Opioid treatment programs	−39.4 (−40.9, −37.9)***	−36.6 (−38.1, −35.2)***	−34.5 (−36.1, −33.0)***
Residential SUD treatment programs	0.3 (−0.2, 0.8)	3.1 (2.6, 3.6)***	5.2 (4.9, 5.5)***
Long Island			
Outpatient SUD treatment programs	3.9 (3.7, 4.2)***	1.5 (1.3, 1.7)***	9.0 (8.7, 9.2)***
Opioid treatment programs	−5.7 (−6.1, −5.4)***	−8.2 (−8.5, −7.8)***	−0.7 (−1.0, −0.4)***
Residential SUD treatment programs	−1.8 (−2.2, −1.5)***	−4.3 (−4.6, −3.9)***	3.2 (2.8, 3.6)***
Mid Hudson			
Outpatient SUD treatment programs	1.1 (0.7, 1.4)***	3.7 (3.4, 4.1)***	12.4 (11.9, 12.8)***
Opioid treatment programs	−12.6 (−13.1, −12.0)***	−9.9 (−10.4, −9.5)***	−1.3 (−1.7, −0.8)***
Residential SUD treatment programs	−7.2 (−7.7, −6.7)***	−4.6 (−5.1, −4.0)***	4.1 (3.5, 4.7)***
Mohawk Valley			
Outpatient SUD treatment programs	−1.7 (−2.8, −0.6)**	−2.6 (−3.5, −1.7)***	16.6 (15.2, 18.1)***
Opioid treatment programs	−15.1 (−16.2, −14.0)***	−16.0 (−17.9, −14.1)***	3.2 (2.0, 4.5)***
Residential SUD treatment programs	10.7 (9.1, 12.2)***	9.8 (8.7, 10.8)***	29.0 (27.2, 30.8)***
North Country			
Outpatient SUD treatment programs	4.2 (2.0, 6.3)***	35.2 (33.0, 37.4)***	41.1 (38.8, 43.3)***
Opioid treatment programs	−59.8 (−63.3, −56.3)***	−28.8 (−31.8, −25.7)***	−22.9 (−25.9, −19.9)***
Residential SUD treatment programs	−23.8 (−26.5, −21.1)***	7.3 (5.1, 9.4)***	13.1 (11.5, 14.8)***
Southern Tier			
Outpatient SUD treatment programs	0.9 (−0.5, 2.2)	22.7 (21.3, 24.1)***	22.5 (21.0, 24.0)***
Opioid treatment programs	−40.5 (−42.2, −38.7)***	−18.6 (−20.4, −16.9)***	−18.8 (−20.6, −17.0)***
Residential SUD treatment programs	−17.6 (−18.5, −16.6)***	4.2 (3.0, 5.5)***	4.1 (3.0, 5.1)***
Western			
Outpatient SUD treatment programs	1.9 (1.6, 2.3)***	4.8 (4.2, 5.3)***	7.0 (6.4, 7.5)***
Opioid treatment programs	−9.6 (−10.2, −9.0)***	−6.8 (−7.2, −6.3)***	−4.6 (−5.1, −4.0)***
Residential SUD treatment programs	−0.9 (−1.5, −0.4)**	1.9 (1.5, 2.3)***	4.1 (3.7, 4.6)***

^a^A positive number for a given facility type (row label) indicates that the robustness (i.e., the mean difference in travel time between the closest and fifth closest facility) is better than for the comparison facility type (column label). A negative number indicates that the robustness of the given facility type is worse than for the comparison facility type. Comparisons are made within economic development regions

^*^P ≤ 0.05; **P ≤ 0.01; ***P < 0.001

*95% CI* 95% confidence interval, *SUD* substance use disorder

**Table 10:** Comparisons of differences in one-way travel time between the closest and fifth closest health care facility, by economic development region, New York State, 2024

		Outpatient SUD treatment programs		Opioid treatment programs		Residential SUD treatment programs	
Economic development region	Population (%)	Difference, minutes (95% CI)^a^	*P*	Difference, minutes (95% CI)^a^	*P*	Difference, minutes (95% CI)^a^	*P*
New York City	8,622,467 (43.1)	Ref		Ref		Ref	
Capital District	1,108,289 (5.5)	− 1.1 (− 1.7, − 0.5)	< 0.001	− 1.4 (− 1.9, − 0.8)	< 0.001	− 1.1 (− 2.1, − 0.2)	0.02
Central New York	781,620 (3.9)	− 0.1 (− 1.0, 0.8)	0.78	13.6 (12.9, 14.3)	< 0.001	− 2.9 (− 3.8, − 2.0)	< 0.001
Finger Lakes	1,219,052 (6.1)	− 0.7 (− 1.2, − 0.1)	0.02	33.9 (32.8, 35.0)	< 0.001	− 6.1 (− 6.7, − 5.4)	< 0.001
Long Island	2,913,593 (14.6)	− 3.1 (− 3.4, − 2.8)	< 0.001	1.1 (0.7, 1.6)	< 0.001	− 3.0 (− 3.5, − 2.6)	< 0.001
Mid Hudson	2,391,754 (12.0)	− 0.4 (− 0.8, − 0.06)	0.02	7.8 (7.2, 8.4)	< 0.001	2.2 (1.7, 2.7)	< 0.001
Mohawk Valley	483,900 (2.4)	14.0 (13.3, 14.7)	< 0.001	22.0 (20.6, 23.4)	< 0.001	− 4.1 (− 5.1, − 3.1)	< 0.001
North Country	422,507 (2.1)	25.1 (23.5, 26.8)	< 0.001	83.7 (79.7, 87.7)	< 0.001	47.4 (44.6, 50.1)	< 0.001
Southern Tier	636,020 (3.2)	9.4 (8.2, 10.6)	< 0.001	45.3 (43.4, 47.2)	< 0.001	22.1 (21.0, 23.2)	< 0.001
Western	1,415,124 (7.1)	0.7 (0.06, 1.3)	0.03	6.8 (5.8, 7.8)	< 0.001	−2.2 (−3.1, −1.3)	< 0.001

^a^A positive number for a given economic development region indicates that the robustness (i.e., the mean difference in travel time between the closest and fifth closest facility) for that facility type is worse, compared with New York City. A negative number indicates that the robustness is better, compared with New York City

*95% CI* 95% confidence interval, *SUD* substance use disorder

## Discussion

In a cross-sectional study, we analyzed travel times and robustness of spatial accessibility of SUD treatment programs, compared with other health care facilities. We found that the statewide spatial accessibility of outpatient SUD treatment programs was similar to or better than FQHCs, dialysis facilities, and hospitals. Compared with NYC, travel times were typically shorter and robustness was typically better in non-NYC urban areas. In contrast, travel times were longer and robustness worse in rural areas.

To contextualize our travel time estimates, they can be compared to travel time standards from government agencies such as state Medicaid programs and the US Veterans Health Administration (VA). Of 35 state Medicaid programs in a recent survey, 13 had SUD travel time standards for managed care plans (median: 30 min, range: 20 to 110 min) [49]. In the VA system, individuals outside a 30 min travel time to mental health care services or a 60 min travel time to specialty services are potentially eligible for care with a non-VA community provider that is more conveniently located [50]. For NYS residents in both urban and rural areas, access to outpatient SUD treatment programs, opioid treatment programs, and residential SUD treatment programs is generally within these other agencies’ published travel time standards.

Compared with residential SUD treatment programs, the spatial accessibility of outpatient SUD treatment programs and OTPs is likely to be more salient to the patient’s experience due to the need to visit the program repeatedly. For these latter programs, rural areas had more limited spatial accessibility than urban areas. However, recent policy changes and funding streams offer opportunities to bridge this gap. For example, efforts to expand telehealth could potentially improve access in rural areas [51]. In a recent study, expanded telehealth flexibilities were associated with higher retention in care and reduced medically treated overdoses [52]. Further, revised federal regulations allow for increased flexibility methadone dosing and in establishing mobile medication units for OTPs, which can increase the reach of methadone treatment, particularly in rural areas [53, 54]. Finally, opioid settlement funds represent a tremendous resource that can be used to invest in access to SUD treatment in rural areas, for example in mobile units that have a high startup cost but can then be sustainable once implemented.

Analyses such as the current one can play a pivotal role in informing the policymaking process in at least two ways. First, identification of areas where spatial accessibility is a barrier to treatment can be used in focused efforts to increase access. For example, NYS has made available over $26 million to expand OTP services in the state [55–58]. Second, analyses of spatial accessibility can allow agencies to evaluate changes in spatial accessibility over time, due to policy interventions, strategic investments, and naturalistic factors such as market forces. Future research can also add other dimensions to a spatial analysis such as service quality, population specialization (e.g., adolescents, LGBT + individuals), and conditions treated (e.g., gambling disorder).

Our study has several limitations. First, our focus of this analysis is NYS and so our findings may not be generalizable outside of the state. Second, while we calculated drive times outside of NYC and public transit times in NYC, there are likely to be residents that travel by public transit (e.g., bus) outside of NYC and by car within NYC. Therefore, our estimates may be biased in either direction. Third, as our focus was on specialty SUD treatment programs, we did not include office-based opioid treatment providers. Including these providers would likely increase estimates of the spatial accessibility of treatment. Fourth, our analysis did not address features such as Americans with Disabilities Act compliance so our estimated travel times may not be representative for individuals with disabilities. Fifth, based on previous research, we estimated travel times with an 8:00 am departure time which may result in overestimates of travel times possible at other times of day (i.e., outside of rush hour). Sixth, our measure of urban status only required the presence of one urban block and so block groups classified as urban represent a range of urban densities. Finally, we did not have sufficient data on patient capacity of facilities to estimate more comprehensive measures of accessibility [59].

In conclusion, we found substantial spatial accessibility of SUD treatment in NYS. While spatial accessibility was more limited in rural areas compared with urban areas, spatial accessibility in rural areas for most people was within travel time standards established by other government agencies. A statewide analysis of spatial accessibility of SUD treatment, compared with other health care facilities, can yield insights into the treatment landscape.

## Data Availability

The datasets analysed during the current study are available from the corresponding author on reasonable request.
